# Supplementary material for: Increased intracellular crowding during hyperosmotic stress
Source: Sci Rep. 2023 Jul 22;13:11834. doi: 10.1038/s41598-023-39090-w (PMC10363123; doi:10.1038/s41598-023-39090-w)
Supplement: Supplementary file 2 — Supplementary Information 2. [file 41598_2023_39090_MOESM2_ESM.pdf]

Supplemental Material

Increased intracellular crowding during hyperosmotic stress

Akira Kitamura[1*], Sho Oasa[2], Haruka Kawaguchi[1], Misato Osaka[1], Vladana Vukojević[2], Masataka Kinjo[1]

1 Laboratory of Molecular Cell Dynamics, Faculty of Advanced Life Science, Hokkaido University, Sapporo, Japan
2 Department of Clinical Neuroscience (CNS), Center for Molecular Medicine (CMM), Karolinska Institutet, Stockholm 17176, Sweden

*To whom correspondence may be addressed. Email: akita@sci.hokudai.ac.jp

Further information about multi-point fluorescence lifetime imaging microscopy (MP-FLIM)

The home-built massively parallel FLIM (mpFLIM) system has been described in our published work Oasa *et al.* ***Anal. Chem.*** 2021 93(35):12011-12021 and related Supplementary Materials (https://www.ncbi.nlm.nih.gov/pmc/articles/PMC8427561/). Briefly, our instrument is equipped with a 488 nm Picosecond Pulsed Diode Laser, PDL 800-D (PicoQuant, Germany), laser pulse width and repetition rate of 68 ps and 50 MHz, respectively. A laser pulse is triggered by the SPC^3 single-photon avalanche diode (SPAD) camera (MPD, Italy), after which the gate is opened and eGFP fluorescence is detected. The gate signal is generated synchronized with the internal 50 MHz (20 ns) reference clock of the camera, and the gates' timing is accurately determined by the internal clock. To optimize the gate width (smallest available value 220 ps) and step duration (smallest available value 20 ps), ATTO488 in aqueous buffer solution was used as a fluorescence lifetime standard, $\tau_{f,ATTO488}$ = 4.16 ns. Gate width and step duration were varied and values for which optimal trade-off between the signal-to-noise and fluorescence lifetime accuracy are obtained were selected, taking into account the convolution of the Instrumental Response Function (IRF) with the intensity measured at the first time gate (Supplemental Figure S9). Based on this, a gate width, Δt_{gate} = 2 ns, step size, Δt_{shift} = 0.2 ns, integration time t_i = 46 ms and a total sampling of 80 points, were chosen to acquire a full FLIM curve. The entire FLIM curve in each pixel is thus recorded by shifting the window frame by Δt_{shift} = 0.2 ns time-steps, where the first window begins at start shift t_s = 2 ns, the second at 2.2 ns, and so on. Eighty measurement points were recorded for each FLIM curve, extending over a 2-18 ns sub-period within a single 20 ns FLIM measurement time defined by the internal clock of the MPD SPC^3 camera. Photons detected within time gate Δt_{gate} = 2 ns were integrated for 46 ms, yielding a total acquisition time of a single FLIM curve of 3.7 s for 80 data points.

To collect fluorescence, a filter set for the enhanced green fluorescent protein (eGFP) was used consisting of an excitation bandpass filter EX BP470/40 nm (central wavelength/band width), long pass dichroic mirror with a cut-off wavelength of 495 nm, and an emission band pass filter EM BP 525/50 (all from Carl Zeiss). Polarizers were not included in the instrument.

Background count rate outside of cells (in the cell culture medium) and the lowest signal observed (Figure 1D) were 0.09 kHz and 27 kHz, thus the signal-to-background ratio (SBR) is > 300. Binning of fluorescence signal in the data analysis was not applied. In our gated method, instrumental response function (IRF, grey line) is shown in Figure S9A. We have tested in the previous paper that single-exponential fitting (mpFCS/FLIM analysis software) and fitting with IRF (DecayFit software) are no changes in 1 – 8 ns fluorescence lifetime and both are in good agreement with values reported in literatures (Supplemental Figure S9B). We, therefore, didn't use IRF for data fitting in this paper. In our fitting analysis on FLIM curves, standard error of mean (SEM) of fluorescence lifetime in each data fitting was 0.017 – 0.03 ns, and the chi-squared values per degrees of freedom are around 2.8×10^{-4}, suggesting high confidence and high signal-to-noise ratio in FLIM curve fitting (R^2 values around 0.999).
